# Extensins: Self-Assembly, Crosslinking, and the Role of Peroxidases

**DOI:** 10.3389/fpls.2021.664738

**Published:** 2021-05-14

**Authors:** John W. Mishler-Elmore, Yadi Zhou, Abhijit Sukul, Mercedes Oblak, Li Tan, Ahmed Faik, Michael A. Held

**Affiliations:** ^1^Department of Chemistry and Biochemistry, Ohio University, Athens, OH, United States; ^2^Complex Carbohydrate Research Center, University of Georgia, Athens, GA, United States; ^3^Interdisciplinary Program in Molecular and Cellular Biology, Ohio University, Athens, OH, United States; ^4^Department of Environmental and Plant Biology, Ohio University, Athens, OH, United States

**Keywords:** extensin, glycoprotein, peroxidase, crosslinking, self-assembly

## Abstract

The extensin (EXT) network is elaborated by the covalent intermolecular crosslinking of EXT glycoprotein monomers, and its proper assembly is important for numerous aspects of basic wall architecture and cellular defense. In this review, we discuss new advances in the secretion of EXT monomers and the molecular drivers of EXT network self-assembly. Many of the functions of EXTs are conferred through covalent crosslinking into the wall, so we also discuss the different types of known intermolecular crosslinks, the enzymes that are involved, as well as the potential for additional crosslinks that are yet to be identified. EXTs also function in wall architecture independent of crosslinking status, and therefore, we explore the role of non-crosslinking EXTs. As EXT crosslinking is upregulated in response to wounding and pathogen infection, we discuss a potential regulatory mechanism to control covalent crosslinking and its relationship to the subcellular localization of the crosslinking enzymes.

## Introduction

Plant cell walls form the basis of plant stature and morphology. Cell walls are complex supramolecular structures composed mostly of carbohydrate, lignin, and glycoprotein polymers. Plant cells produce two types of cell walls: a thinner primary wall that is deposited as plant cells grow and elongate, and a thicker secondary wall that accumulates after cell growth ceases. As these walls are produced, individual cell wall polymers undergo molecular assembly to form three-dimensional composite networks with specific physiochemical properties tailored to individual cell types. Exactly how these networks are assembled remains a bit of a mystery.

Many cell wall polymers become crosslinked into the wall. Crosslinking can impart architectural stabilization for normal wall reinforcement, but also to build physical barriers in response to pathogen infection or wounding. Here, we use the term “crosslink” in a broader sense to include both non-covalent and covalent interactions among cell wall polymers to form interacting networks that stabilize wall architecture (Fry, 1986). Crosslinks can be divided into two categories: homopolymeric crosslinks and heteropolymeric crosslinks. The association of glucan chains by hydrogen bonding (Atalla and Vanderhart, 1988; Delmer and Amor, 1995; Haigler et al., 2016), the crosslinking of hemicellulose polymers by xyloglucan endotransglucosylase/hydrolases (Rose et al., 2002), Ca^+2^ crosslinking of homogalacturonans, and extensin (EXT) crosslinking by covalent tyrosine linkages (Held et al., 2004) may all be considered as examples of homopolymeric crosslinking. Various types of heteropolymeric crosslinks have also been described. For example, linkages between hemicellulose and cellulose microfibrils (Bauer et al., 1973; Hayashi, 1989; Carpita and Gibeaut, 1993; Dick-Pérez et al., 2011), pectin and cellulose (Dick-Pérez et al., 2011), pectin and hemicellulose with arabinogalactan protein (AGP; Tan et al., 2013), EXT and pectin (Qi et al., 1995; Nunez et al., 2009), AGP with pectin (Hijazi et al., 2014a), cinnamic acid derivatives with hemicelluloses (Fry, 1986; Mueller-Harvey et al., 1986; Ishii, 1991; Ishii and Tobita, 1993), and cinnamic acid derivatives and lignin (Ralph et al., 1995; Jung, 2003) have all been demonstrated. Further, linkages between AGP and EXT (Tan et al., 2018), cinnamic acid derivatives and pectin side chains (Levigne et al., 2004), and EXT with lignin (Cong et al., 2013) have also been suggested. Thus, it is plausible that any given wall polymer has at least one type of crosslink with the other cell wall polymers. Having multiply interconnected polymeric networks (hemicellulose, pectin, and structural glycoproteins) may facilitate load sharing in response to turgor-driven wall extension by increasing stress distribution (Park and Cosgrove, 2012). Identification of these crosslinks has been difficult, in part due to their relatively low abundance within the wall, making them proverbial “needles in a haystack.” The wide variety of wall polymers and diversity of known crosslinks to date also suggest that there are most certainly other crosslinks yet to be identified.

One of the best-studied cases of homopolymeric cell wall polymer crosslinking involves a class of hydroxyproline-rich glycoproteins (HRGPs) known as EXTs. EXT crosslinking is necessary for general wall architecture, integrity, and development (Sadava and Chrispeels, 1973; Goodenough and Heuser, 1985; Goodenough et al., 1986; Hong et al., 1989; Knox et al., 1991; Pennell et al., 1991; Goldman et al., 1992) and also plays specific physiological roles in positioning the cell plate for the first asymmetric division during embryogenesis (Hall and Cannon, 2002; Cannon et al., 2008), pollen tube growth (Mecchia et al., 2017; Fabrice et al., 2018; Sede et al., 2018), root hair growth (Baumberger et al., 2001, 2003; Velasquez et al., 2011), mechanical wounding (Chen and Varner, 1985; Showalter et al., 1991; Hirsinger et al., 1999; Salvà and Jamet, 2001; Merkouropoulos and Shirsat, 2003; Guzzardi et al., 2004), and stress and pathogen responses (Hammerschmidt et al., 1984; Showalter et al., 1985; Mazau and Esquerré-Tugayé, 1986; Lawton and Lamb, 1987; Bradley et al., 1992; Merkouropoulos and Shirsat, 2003).

Many of the functions of EXTs are a result of their intermolecular interactions and crosslinking properties in the wall. Through these interactions, EXTs are thought to help template primary wall architecture as well as participate in the cessation of primary wall formation. Here, we focus on a discussion of the literature surrounding the self-assembly and covalent crosslinking of the EXT structural glycoprotein network. After providing a brief discussion of EXT structure and composition, we take a look at recent advances in cell wall glycoprotein analyses and the enzymes responsible for the covalent crosslinking of EXTs.

## The HRGP Superfamily: A Brief Overview on Classification, Post-Translational Modification, and Secretion

### HRGP Classifications

**Figure 1:**
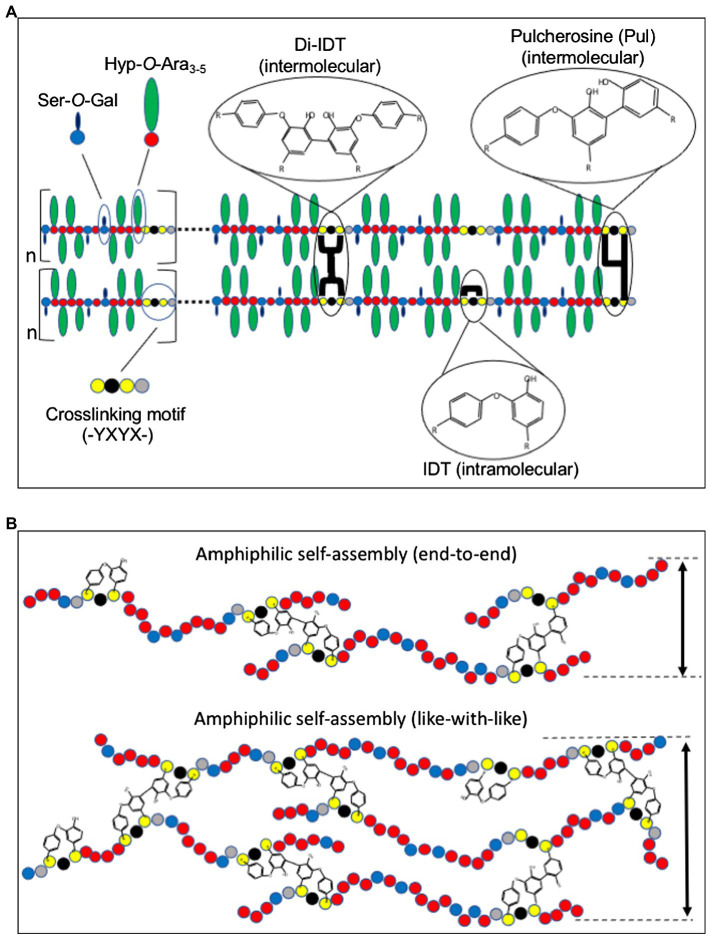
Model for extensin (EXT) network assembly and crosslinking. **(A)** EXT precursors self-align through amphiphilic interactions between alternating hydrophilic Hyp-glycomodules (Hyp-*O*-Ara_3-5_ and Ser-*O*-Gal) and hydrophobic crosslinking motifs (-Tyr-X-Tyr-X). Intermolecular crosslinks such as pulcherosine (Pul) and di-isodityrosine (di-IDT) and intramolecular crosslinks *via* isodityrosine (IDT) are indicated. Intermolecular crosslinks are catalyzed by extensin peroxidases (EPs) and result in insolubilization of EXT precursors in the cell wall. **(B)** Schematic presentation of “end-to-end” and “like-with-like” arrangements during amphiphilic self-assembly of EXTs (Cannon et al., 2008). While “end-to-end” arrangement may favor branching and network elongation, “like-with-like” self-assembly may contribute to the thickness of the EXT polymer. Amino acids are indicated as colored circles: Tyr (yellow), Hyp (red), galactosylated Ser (blue), and variable amino acids (X, black and gray).

Structural glycoproteins of the plant cell wall are both structurally and functionally diverse and most belong to the HRGP superfamily, which consists of a broad continuum of glycoproteins. HRGPs can be loosely categorized into subclasses, ranging from the heavily glycosylated AGPs to the lightly glycosylated proline-rich proteins (PRPs). Intermediate to these two HRGP subgroups are the network-forming EXT glycoproteins. Our discussion here focuses on the classical EXTs, because they tend to have the highest levels of molecular symmetry, which is important for wall scaffolding, and are enriched in crosslinking motifs (Liu et al., 2016), which means they are able to undergo covalent crosslinking to form the polymeric EXT wall network. A defining structural feature of classical EXTs is that they possess EXT domains that are characterized as having multiple [-Ser-Hyp_3-5_-]_n_ peptide repeats (Kieliszewski and Lamport, 1994; Kieliszewski, 2001). These repeat [-Ser-Hyp_3-5_-]_n_ modules are frequently interspaced by a variety of tyrosine (Tyr)-containing crosslinking motifs (esp. -Tyr-X-Tyr-; Figure 1A), which are important for EXT self-assembly and covalent crosslinking within the wall. Covalent EXT crosslinking by specialized class III peroxidases known as extensin peroxidases (EPs) results in the insolubilization of EXTs and limits their extraction from the wall. While many classical EXT precursors are covalently crosslinked into the wall, a portion of EXT precursors remains ionically associated with the wall but is non-covalently crosslinked (NCL EXTs). These NCL EXT precursors may play an equally important role in stabilizing wall networks (discussed further below).

Liu et al. (2016) surveyed 16 plant and related algal genomes for the presence of EXTs. Here, they defined classical EXTs as having three or more blocks of [-Ser-Hyp_3-5_-]_n_ peptide repeats (-Tyr-X-Tyr-) crosslinking motifs, and an N-terminal signal sequence that guides them to the secretory pathway. They found that while classical EXTs are abundant in higher dicot genomes, they are missing from non-vascular model plant genomes, indicating that they arose with land plant evolution. Furthermore, classical EXTs are notably not present in gymnosperms [with the lone exception of Norway spruce (*Picea abies*)] and monocots (such as *Zea mays*, *Oryza sativa*, and *Brachypodium distachyon*; Liu et al., 2016). This is surprising given their abundance and important physiological roles in dicot plants. Monocots do however contain EXT-like precursors that are rich in threonine (dubbed THRGPs), but they do not technically fit into the classical EXT category and are not known to be covalently crosslinked (Kieliszewski et al., 1990; Schnabelrauch et al., 1996). It has been suggested that monocots may make up for the absence of classical EXTs by the analogous crosslinking of glucuronoarabinoxylan polymers *via* diferulate bridges that are especially abundant in grass cell walls (Mnich et al., 2020). Further work is needed to understand the nature of the EXT network in grasses.

### Post-translational Modification of Classical EXTs

**Figure 2:**
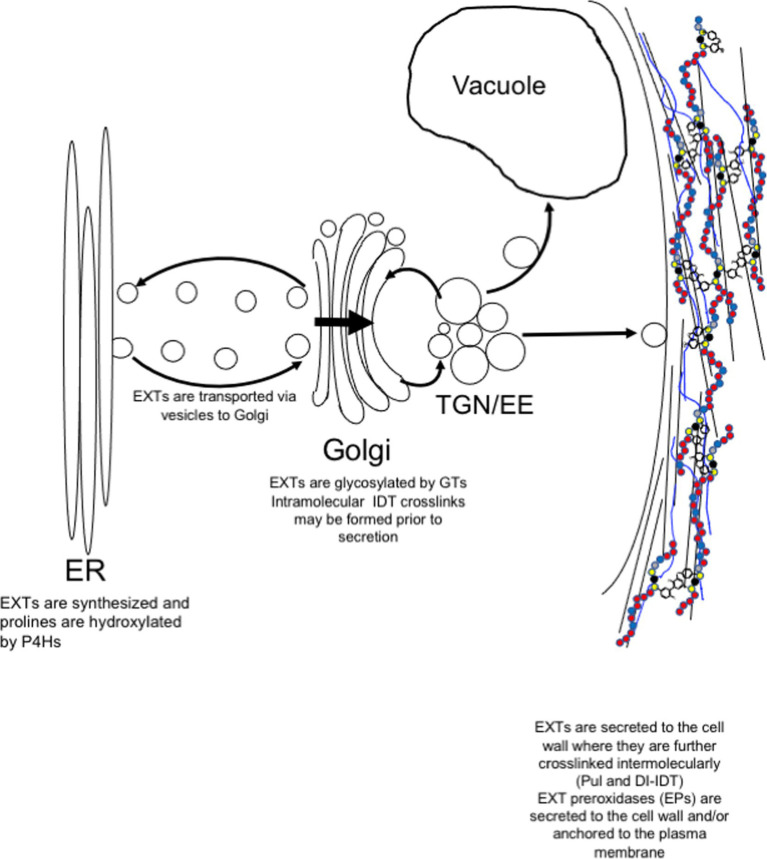
Post-translational modification and secretion of EXTs. EXTs are synthesized in the endoplasmic reticulum (ER) and post-translationally modified by prolyl 4-hydroxylases (P4Hs). In the Golgi, EXTs are further modified by Hyp-*O*-arabinosylation and Ser-*O*-galactosylation. EXTs are sorted in trans-Golgi network (TGN) for secretion to the plasma membrane. Somewhere between the TGN and the cell wall, intramolecular IDT crosslinks are formed before release to the cell wall where they self-associate to form the EXT network. EXT monomers can become covalently crosslinked by extensin peroxidases (EPs). EPs use extracellular hydrogen peroxide to form covalent intermolecular crosslinks, such as Pul and di-IDT, which covalently lock EXTs within the cell wall.

EXTs are heavily post-translationally modified proteins. Signal sequences direct ribosomes translating EXTs to the endoplasmic reticulum (ER) for EXT entry into the secretory pathway (Figure 2). As they transfer into the ER, EXT precursors are first modified by proline hydroxylation, which is catalyzed by specific prolyl-4-hydroxylase isozymes (P4Hs, EC 1.14.11.2). P4Hs use O_2_, 2-oxoglutarate, and Fe^+2^ and ascorbate cofactors to catalyze the formation of *trans*-4-hydroxyproline (Hyp) from proline. P4Hs are type II membrane proteins that localize to both the ER and the Golgi membranes (Yuasa et al., 2005; Velasquez et al., 2015). In Arabidopsis, three of the 13 predicted P4H family members, namely, P4H5, P4H2, and P4H13, are thought to interact to form protein complexes along the ER/Golgi pathway that are specifically important for hydroxylating the prolines of [-Ser-Pro_3-5_-]_n_ EXT motifs (Velasquez et al., 2015). In root hairs, P4H5 appears to be a central, non-redundant player in these complexes and has been shown to preferentially hydroxylate the first three prolines in a -Ser-Pro-Pro-Pro-Pro-motif to produce -Ser**-Hyp-Hyp-Hyp-**Pro- *in vitro*. P4H2 and P4H13 are thought to play redundant roles by subsequently hydroxylating the C-terminal proline of the -Ser-Hyp-Hyp-Hyp-Pro- (forming -Ser-Hyp-Hyp-Hyp-**Hyp**-; Velasquez et al., 2015). Biochemical data certainly support P4H2 having a preference for hydroxylating the ultimate proline in EXT motifs (Tiainen et al., 2005). Given its genetic redundancy with P4H2, the same role is assumed for P4H13.

After hydroxylation, Hyp residues of EXTs can become *O*-glycosylated (Figure 2; Lamport and Miller, 1971). The type of glycosylation is prescribed by the primary sequence of each HRGP in accordance with the “Hyp-contiguity” hypothesis (Kieliszewski and Lamport, 1994). With few exceptions (Ohyama et al., 2009; Ogawa-Ohnishi et al., 2013), contiguous Hyp residues (i.e., [-Ser-Hyp_4_-]_n_ which are common in EXTs) prescribe attachment of short oligoarabinosides (Hyp-*O*-Ara_3-5_), whereas repetitive, non-contiguous Hyp residues (i.e., [-Ser-Hyp-]_n_ common to AGPs) are sites for *O*-linked type II arabinogalactan polymer attachment (Shpak t al., 2001; Tan et al., 2003; Held et al., 2004). Several arabinosyltransferases have been shown to synthesize Hyp-*O*-oligoarabinosides from UDP-L-Ara*f*, including hydroxyproline-*O*-β-arabinosyltransferase 1-3 (HPAT1-3; Ogawa-Ohnishi et al., 2013), reduced residual arabinose (RRA1-3; Egelund et al., 2007; Velasquez et al., 2011), xyloglucanase 113 (XEG113; Gille et al., 2009), and extensin arabinose deficient (ExAD; Møller et al., 2017). Serine residues of EXTs can also be monogalactosylated by serine-galactosyltransferase 1 (SGT1; Saito et al., 2014). These enzymes are highly coordinated at the gene expression level (Marzol et al., 2018) and are present at the same time in the Golgi proteome (Nikolovski et al., 2014), suggesting the potential for multi-glycosyltransferase (GT) complex formation. These and other cell wall GTs have been adequately reviewed elsewhere (Velasquez et al., 2012; Held et al., 2014).

### Extensin Secretion to the Apoplast

After hydroxylation in the ER and Golgi and *O*-glycosylation in the Golgi, EXTs are secreted to the apoplast as soluble monomeric precursors (Figure 2). Details concerning secretion machinery and vesicle contents have been murky. However, recent data may shed some light on this process and indicate that post-Golgi secretion of EXTs may be mediated by the exocyst complex (Beuder et al., 2020). The exocyst complex is a highly conserved secretory complex found in all eukaryotic cells and is composed of Sec3, Sec5, Sec6, Sec8, Sec10, Sec15, Exo70, and Exo84 proteins (TerBush et al., 1996; Guo et al., 1999), of which Exo70A appears to be among the most abundant and plays a central role (Synek et al., 2006). With the help of Arf, Rab, Rho GTPases, and SNARE proteins, the exocyst acts as a tethering complex for the polarized delivery of post-Golgi secretory vesicles to the plasma membrane (PM). Exocyst complex components are critical for cell growth, especially for cells undergoing tip growth, like pollen tubes (Cole et al., 2005; Hála et al., 2008) and root hairs (Wen et al., 2005; Synek et al., 2006), but also for seed coat mucilage deposition (Kulich et al., 2010) and pistil-pollen compatibility interactions (Samuel et al., 2009). The exocyst complex has also been shown to help mediate the delivery of important wall components to the PM, like PM-resident reactive oxygen species (ROS)-generating enzymes (e.g., NADPH oxidase; Markovic et al., 2020) and cell wall biosynthetic enzymes (e.g., cellulose synthases; Zhu et al., 2018), as well as secreted cargo, like pectins (Kulich et al., 2010). Cellulose synthases have also been shown to be delivered in vesicles containing syntaxin of plants 61 (SYP61; Drakakaki et al., 2012). It is not clear whether these SYP61-containing vesicles co-localize with those of the exocyst complex at present.

Mutation of *Hpat1* and *Hpat3* abolishes Hyp *O*-arabinosylation of EXTs and causes severe pollen tube defects at the growing tip and reduced fertility (MacAlister et al., 2016). A suppressor screen of *hpat1/3* double mutants, which displays under-arabinosylation of EXT Hyp residues, showed that a mutation in *Exo70a2* restored pollen tube growth and male transmission (Beuder et al., 2020). As Exo70A2 is critical for exocyst-mediated delivery of post-Golgi vesicles to the PM, the most likely explanation is that reduced secretion in *exo70A2/hpat1/hpat3* triple mutants prevents un-glycosylated HRGPs from being delivered to the cell wall. These data are important for two reasons: (1) it strongly suggests that EXT secretion is mediated by the exocyst complex and (2) secretion of mis-or underglycosylated HRGPs may interfere with normal EXT functions and can have serious negative impacts on wall architecture, supporting their role in templating/scaffolding other wall polymers. While the data strongly imply that EXTs might be trafficked through this pathway, it would be interesting to see whether this extends more broadly to other HRGPs. It is unclear how this relates to cysteine endoproteases (CEPs) that may be involved in quality control for mis-or underglycosylated EXTs (Helm et al., 2008; Marzol et al., 2018). One would presume these CEPs are activated/upregulated in *hpat* mutants, but this remains to be tested.

## Extensin Network: Self-Assembly and Crosslinking

### Non-Covalent Self-Assembly of EXTs and the Role of Amphiphilicity

Like most other non-cellulosic cell wall polymers, EXT precursors are secreted to the apoplast of cells where they are thought to self-associate prior to wall incorporation (Cannon et al., 2008). Multiple non-covalent interactions, including hydrophilic interactions between Hyp-*O*-arabinosides and hydrophobic interactions between regularly spaced crosslinking motifs, play a role in driving self-assembly (Figure 1A; Cannon et al., 2008; Chen et al., 2015). It is hypothesized that the amphiphilic nature of EXTs is a major driver of non-covalent molecular self-assembly (Cannon et al., 2008; Lamport et al., 2011).

Perhaps, the most visually striking support for EXT self-alignment was the use of atomic force microscopy (AFM) to examine root-shoot-hypocotyl (RSH) EXT precursor assembly *in vitro* (Cannon et al., 2008). Purified RSH monomers were allowed to self-assemble on a graphite surface prior to AFM imaging in surface-scanning mode. RSH monomers spontaneously assembled into complex, yet organized dendritic networks with subunit structures approximately the size of individual RSH monomers (~130 nm). This was observed in the absence of EP, the enzyme responsible for covalently crosslinking EXTs, indicating that covalent crosslinking itself is not needed to form complex EXT molecular scaffolds, but rather EPs may come along later to “lock” the scaffold into place. Somewhat surprising was the absence of these dendritic structures using purified, synthetic tomato P3 analog EXT monomers (YK20). YK20 monomers are highly periodic (almost palindromic) and have similar molecular size and glycosylation status as RSH (Held et al., 2004). One would have suspected that YK20 would also self-assemble into a dendritic molecular scaffold-like RSH; however, these monomers are associated to produce many individual punctate structures. The authors hypothesized that RSH monomer alignment is more capable of end-to-end assembly which favors the pulcherosine (Pul) crosslinks (Figure 1B), while YK20 alignment prefers to sandwich (described as like-with-like assembly) which favors di-isodityrosine (di-IDT) crosslinks. It was also noted that of the 20 classical EXTs found in Arabidopsis, 18 have C-terminal -(Tyr-X-Tyr)- crosslinking motifs. Additional analysis of AtEXTs (including RSH) by the BIO OHIO 2 platform also shows that many have extreme N-terminal -(Tyr-X-Tyr)- motifs (Liu et al., 2016). The abundance of both N-and C-terminal crosslinking motifs in Arabidopsis classical EXTs may favor more end-to-end alignment and crosslinking and therefore may also lead to more RSH-like assembly. It would be interesting to test whether N-and C-terminal crosslinking motifs favor end-to-end linkage, whereas internal crosslinking motifs favor side-by-side association using designer EXT analogs that manipulate the position and density of the crosslinking motifs.

Another player in EXT self-alignment is the oligoarabinosides *O*-linked to Hyp residues. Proper glycosylation of EXT precursors is necessary for self-assembly and subsequent covalent crosslinking. This is supported by the fact that mutation of either GTs needed for glycosylation of EXTs or their corresponding prolyl hydroxylases leads to severe root hair growth abnormalities (Velasquez et al., 2011) and issues associated with plant defense (Tan and Mort, 2020). The idea being here that loss of Hyp and/or inadequate glycosylation of EXTs directly leads to problems with wall assembly which translates to wall growth defects and increases in pathogen susceptibility *in vivo*. Additionally, numerous *in vitro* studies have been conducted to assess the role of EXT Hyp-oligoarabinosides for crosslinking. Complete removal of Hyp-*O*-arabinosides by deglycosylation of monomeric precursors using anhydrous hydrogen fluoride (HF; Mort et al., 1989) prior to crosslinking blocks polymerization for AtEXT3 (RSH; Chen et al., 2015), the synthetic P3-analog EXT YK20 (Held et al., 2004), and native tomato P1 EXT (Schnabelrauch et al., 1996). Further, partial de-arabinosylation by limited acid hydrolysis of these substrates lowered their crosslinking rates in a manner dependent with glycan content, demonstrating the necessity of the oligoarabinosides for EXTs crosslinking (Chen et al., 2015). Together, these data strongly imply that Hyp-*O*-arabinosides also play a fundamental role in molecular self-assembly of EXT monomers.

### Covalent Crosslinking of EXTs

EXT crosslinking is mediated by covalent crosslinking of Tyr residues. These Tyr residues are part of crosslinking modules/motifs that are regularly spaced along the backbone of classical EXTs. The first evidence for Tyr-based crosslinks came with the discovery of isodityrosine (IDT) in EXT hydrolysates (Figure 1A; Fry, 1982). IDT was originally thought to mediate EXTs intermolecular crosslinking, and its formation would limit cell elongation (Lamport, 1970; Fry, 1979); however, it was later shown that IDT was only detected as EXT intramolecular crosslinks (Epstein and Lamport, 1984). Early work suggested the involvement of specific EPs for the formation of IDT and insolubilization of EXT into the wall (Fry, 1979). It was later demonstrated that EXT and PRP precursors that lacked Tyr-containing motifs could not be crosslinked *in vitro* by an EP from tomato (discussed below), leading the authors to conclude that Tyr-containing motifs were necessary for EXTs crosslinking (Schnabelrauch et al., 1996). Not long after this work, higher-ordered Tyr-based crosslinks, di-IDT (Brady et al., 1996) and Pul (Brady et al., 1998; Figure 1A), were discovered in the acid hydrolysates of tomato cell wall preparations. Subsequent work showed that the abundance of these Tyr-containing oligomers increased with conditions that stimulated EXTs crosslinking *in vivo* such as fungal elicitation and peroxide treatment (Brady and Fry, 1997). *In vitro* crosslinking studies using recombinant EXT precursors produced from synthetic genes showed that a pI 4.6 tomato EP specifically catalyzed the crosslinking of EXT monomers containing -Tyr-X-Tyr-motifs by the formation of di-IDT (Held et al., 2004). EXT monomers that lacked -Tyr-X-Tyr-motifs did not crosslink, highlighting the importance of Tyr residues in the crosslinking motif. Further analysis of the crosslinked EXT polymer showed the presence of di-IDT (which was not detected in soluble YK20 monomers) as well as concomitant reductions in both IDT and Tyr. These data were the first to indicate the nature of EXT intermolecular crosslinks.

### *In vitro* vs. *in vivo* Crosslinking

Subsequent studies have cautioned the use of *in vitro* studies for inferring the formation of Pul and di-IDT crosslinks *in vivo* (Cegelski et al., 2010; Hijazi et al., 2014b), and we agree. Just because Pul and di-IDT are formed by native EPs *in vitro* does not mean they are formed in native walls, despite the isolation of Pul and di-IDT crosslinking amino acids (Brady et al., 1996, 1998). However, very recent wall proteomic data now allow us to make that judgment. Both Pul and di-IDT-linked EXT peptides have been recently detected using advanced LC–MS/MS-based wall proteomic techniques that are able to predict and detect crosslinked EXT peptides, thus finally demonstrating that both Pul and di-IDT do indeed serve as EXT intermolecular crosslinks *in vivo* (Gainey, 2020).

## Extensin Peroxidases

### Class III Peroxidases

EPs are responsible for intermolecular covalent crosslinking of EXT monomers. EPs are CIII Prxs (E.C. 1.11.1.7), which are heme-containing peroxidases directed to the secretory pathway (Welinder, 1992). CIII Prxs are members of large multigene families. The Arabidopsis genome encodes 73 CIII Prxs (Valério et al., 2004). Rice has 138 (Passardi et al., 2004a), and other plant genomes have comparably high numbers (Fawal et al., 2012). Individual CIII Prxs share many functional, structural, catalytic, and post-translational characteristics and are involved in processes like cell wall assembly and differentiation, cell elongation, and pathogen defense (Hiraga et al., 2001; Passardi et al., 2004b; Cosio and Dunand, 2009). CIII Prxs are also known for filling numerous roles including hormone catabolism (Lagrimini et al., 1997; Gazaryan et al., 1999), salt tolerance (Amaya et al., 1999), lignin biosynthesis (Koutaniemi et al., 2005; Zhao et al., 2013; Shigeto and Tsutsumi, 2016), and suberization (Espelie and Kolattukudy, 1985; Bernards et al., 1999; Quiroga et al., 2000; Keren-Keiserman et al., 2004), in addition to EXT crosslinking (Schnabelrauch et al., 1996; Held et al., 2004; Dong et al., 2015). Large gene family sizes, overlapping functions, and functional redundancy have made assigning individual function(s) to specific CIII Prx isoforms quite challenging. Functional prediction by sequence homology to CIII Prxs of known function has also been relatively fruitless due to their low overall primary sequence similarities within the family.

Structurally speaking, CIII Prxs are far more conserved and are characterized as having three conserved α-helices that distinguish them from class I and II peroxidases, as well as distal and proximal heme-binding sites and four di-sulfide linkages made up of eight strictly conserved Cys residues (Welinder, 1992; Hiraga et al., 2001). A region located between proximal heme-binding site and the seventh conserved Cys is variable in both length and sequence, and has been shown to form part of the substrate access channel for both peanut (Schuller et al., 1996) and horseradish peroxidases (Gajhede et al., 1997) and thus likely dictates substrate specificity of the various CIII Prxs (Chittoor et al., 1999; Hiraga et al., 2001). Mechanistically, CIII Prxs can reduce the available H_2_O_2_ (*via* the peroxidative cycle) using various electron donors (e.g., EXT tyrosines, monolignols, ferulic acids, and suberin) leading to crosslinking that is associated with restricting cell elongation. Extracellular peroxide is generally produced by PM-embedded NADPH oxidase isoforms, including the respiratory burst oxidase homologD, which use intracellular NADPH to reduce extracellular O_2_ to form peroxides in the apoplast (Mignolet-Spruyt et al., 2016). Additionally, CIII Prxs can also produce H_2_O_2_ and subsequently ${\mathrm{OH}}^{\cdot }$ 250 radicals (*via* the hydroxylic cycle) to regulate extracellular ROS production for cellular defense and stimulation of cell elongation (Chen and Schopfer, 1999; Schopfer, 2001; Dunand et al., 2003). Assigning specific functions to each CIII Prx isoform has been quite difficult. A combination of 3D structural approaches (e.g., crystallography or *in silico* modeling that specifically examines the active site shape and microenvironment), *in vitro* biochemical activity, subcellular localization, and expression profiling will be necessary to help define substrate preferences for individual isozymes. Despite the wide-ranging functional implications of CIII Prxs, here we focus on EPs, which use the peroxidative cycle *in vitro*.

### Known and Suspected EPs

CIII Prx-catalyzed EXT covalent crosslinking was originally proposed in the 1970s, but it was several years later that it was first demonstrated *in vitro*. Everdeen and colleagues reported their investigations into EXT crosslinking activity observed in calcium chloride eluates of suspension-cultured tomato cells (Everdeen et al., 1988). The eluates were used in crosslinking reactions using isolated EXT precursors and hydrogen peroxide as co-substrates. Reaction products were separated by gel filtration whereby a decrease in EXT precursor corresponded with increases in large molecular mass EXT oligomers. To investigate the linkages holding these new products together, their integrity was tested by treatment with ethylene glycol bis(2-aminoethyl)tetraacetic acid and 2-mercaptoethanol, which failed to disrupt the assembly as observed by electron microscope, indicating that neither calcium crosslinking nor di-sulfide bonds were primary contributors to oligomer assembly. Side-by-side crosslinking reactions, some incubated with catalase and others without, provided proof that the newly found EXT crosslinking enzyme was a peroxidase. Since this work, numerous additional EPs have been reported in tomato (Brownleader et al., 1995; Schnabelrauch et al., 1996; Dong et al., 2015), mustard seed (Magliano and Casal, 1998), lupin (Jackson et al., 1999; Price et al., 2003), French bean (Blee et al., 2001), and grapevine (Jackson et al., 2001; Pereira et al., 2011). Of these, however, only LEP1, FBP1, and the pI4.6 tomato EP (called candidate gene 5, CG5) have been demonstrated to crosslink EXTs *in vitro*. Furthermore, crosslinked products (di-IDT and Pul) have only been characterized from reactions using CG5 (Held et al., 2004).

Peroxidase CG5 was originally demonstrated to possess specificity for EXTs containing tyrosine and lysine [-Val-Tyr-Lys-] crosslinking motifs, and that carbohydrate did not participate in the covalent crosslink it formed (Schnabelrauch et al., 1996). These motifs were common to tomato precursor 1 (P1) peptides. At the time, the full protein sequence of tomato P1 was not known, but we now know that P1 also contains -Tyr-X-Tyr-motifs that are also able to participate in crosslinking (Liu et al., 2016). The group however went on to suggest the possibility of a role for tyrosine and/or lysine being directly involved in the covalent crosslinking for EXTs.

The gene encoding the CG5 peroxidase was eventually identified in 2015 (Dong et al., 2015). Narrowing down bioinformatic results of predicted tomato peroxidases having both signal sequences and predicted pIs near 4.6, eight candidate genes were selected. Proteomics then narrowed the selection to three candidate genes. *In vitro* EXT crosslinking assays using native P1 tomato EXT precursors confirmed the EXT crosslinking activity of CG5 and identified *Solyc02g094180* as the gene encoding the pI 4.6 EP. This work has paved the way for future characterization of the EPs, as well as 3D structural, functional genomics, and expression studies to better understand EPs’ roles in development and plant defense.

Even though only a handful of EPs have been isolated or characterized, more candidates have been identified through bioinformatics and await further investigation. Naturally, Arabidopsis is at the forefront of these efforts, as the classical dicot model organism, and it is somewhat surprising that no EP has been clearly identified in Arabidopsis yet. The bioinformatics resource BIO OHIO identified as many as 32 CIII Prxs in Arabidopsis whose expression profiles are consistent with those of EXT genes (Showalter et al., 2010). Despite this number, only a few have been implicated as actual EPs. AtPrx71 has been shown to produce protein radicals *in vitro* (Shigeto et al., 2014), and overexpression lines saw coincident increases of *AtExt4* expression, suggesting its identity as an EP (Raggi et al., 2015). Mutant lines of *AtPrx*s *9* and *40* show similar phenotypes to *AtExt18* mutants in microspore formation and the tapetum. Further, when co-infiltrated in tobacco leaves, these peroxidases have been demonstrated to crosslink AtExt23 *in vivo* (Jacobowitz et al., 2019).

Three more promising EP candidates have been identified in Arabidopsis as well, due to the work that localized expression of all three CIII Prx genes to root hairs, and phenotypes observed in mutant lines (Mangano et al., 2017). *AtPrx01*, *AtPrx44*, and *AtPrx73* mutant phenotypes suggested roles in ROS homeostasis and root hair growth. Subsequent investigations utilizing gain-of-function experiments to complement the previous work, as well as *in vitro* peroxidase assays, promoter:GFP reporter experiments, and molecular characterization of phenotypes in triple mutant and overexpression lines have continued to build on this line of research (Marzol et al., 2020). And though these AtPrxs are not yet fully characterized, nor can be confirmed as EPs, the evidence gathered strongly suggests their involvement in EXT crosslinking. It will be interesting to test these peroxidases for their *in vitro* EXT crosslinking properties and kinetics.

## Perspectives and Open Questions

### Are EPs Opportunists or Are Their Actions Regulated?

EPs are clearly secreted from suspension-cultured cells as they are readily harvested from cell culture media (Schnabelrauch et al., 1996; Dong et al., 2015). However, the precise location of assembly and crosslinking has not been demonstrated *in planta*. This leaves open several questions: Where are EXTs actually crosslinked? Do EXT self-assembly and crosslinking initiate in the apoplast as they are deposited into the wall, or are they assembled in the wall first then later crosslinked by EPs?

Knowing the *in vivo* localization of EPs may give some clues. Few studies have examined the actual subcellular localization of CIII Prxs *in vivo*, and none have been published for EPs. Inferring the localization of EPs in cell cultures presents problems as well since these cells are constantly under oxidative stress and may represent a constitutively activated state. Proteomic data confirm that of 73 CIII Prxs in Arabidopsis, 32 were detected as extracellular, 17 were detected in PM fractions, and seven were vacuolar (Francoz et al., 2015). The seven vacuolar Prxs all have predicted C-terminal vacuolar transit signals, as would be expected. It is somewhat surprising that 17 out of 56 (or 30% of the non-vacuolar CIII Prxs) were found in PM fractions (Tanz et al., 2012; Francoz et al., 2015), which include leading Arabidopsis candidates, AtPrx1, 44, and 73 (Francoz et al., 2015). PM-associated CIII Prxs have been known for some time (Askerlund et al., 1987; Lüthje et al., 2011), and at least partial PM localization has been previously suggested for CG5 (Dong, 2015; Mishler-Elmore, 2020); thus, it is tempting to speculate PM localization for CG5 and other EPs.

PM localization of EPs may reveal an interesting regulatory mechanism of crosslinking of EXTs in the wall. Indeed, EPs may be localized to the PM where they initiate radical coupling of EXTs as they pass into the apoplast, as seen for monolignol polymerization (Ralph et al., 2004). Another possibility is that EPs are transiently anchored to PM until physiological cues or signals induced EP release into the apoplast for subsequent crosslinking of EXTs (Mishler-Elmore, 2020). This would be more in accordance with their roles in wound and stress response, pathogen infection, and symbiosis. This might also explain their secretion in suspension-cultured cells which can mimic stressed/wounded states. The separation of EXT secretion and crosslinking also permits time for proper self-assembly of other polymers into the appropriate wall structure. What accounts for PM association remains to be determined. Some CIII Prxs have putative TMDs, but others may associate with the PM by uncleaved N-terminal signal peptides that act as transmembrane anchors (Lüthje et al., 2011).

If EPs are transiently anchored in the PM and await a signaling event to perform crosslinking, what might the signal(s) be? ROS generation (Baxter et al., 2014)? Calcium flux (Evans et al., 2001; Lamport and Várnai, 2013)? and Is there a role for pectin fragments (Denoux et al., 2008) and wall-associated kinases (Kohorn and Kohorn, 2012) or other receptor-like kinases like Fei1/Fei2 (Xu et al., 2008), Theseus1 (Hématy et al., 2007) or other wall integrity sensing pathways? These studies remain, and a more thorough subcellular and temporal localization analysis of known and suspected EPs is needed.

### Are There Other Types of EXT Crosslinks?

Tyrosine-based crosslinks are the only known stabilizers of the EXT network in the wall. However, grasses and gymnosperms are relatively poor in EXTs, suggesting their functions have been absorbed by other types of wall crosslinks in these plants. Furthermore, plants like Arabidopsis have relatively low amounts of known crosslinking amino acids IDT, Pul, and di-IDT. Couple this observation with the difficulty in identifying EPs in Arabidopsis and the significant functional redundancy among peroxidases, and one is left with the sense that there may be other ways the EXT network is stabilized. At the same time, this also leaves open the possibility that some plants like Arabidopsis may not actually express (or need) a functionally distinct EP. Therefore, it is important to consider plant genomic models carefully when examining EPs.

As mentioned above, there are two sub-populations of classical EXTs: those that are CL and those that are ionically associated but NCL. NCL EXTs may play an equally important role in stabilizing the EXT network. For example, wall association of LRX1 does not require tyrosine (Ringli, 2010), but does appear to be driven by hydrophobic regions in the EXT domains of LRX1 in accordance with self-assembly principles put forth by Cannon et al. (2008). Other mechanisms of EXT crosslinking must exist, and these areas need to be explored.

A long-standing hypothesis indicates a role for the glycan substituents of EXT as crosslinking targets, especially with an RG-I-like fraction of pectin (Keegstra et al., 1973; Iiyama et al., 1994; Qi et al., 1995; Nunez et al., 2009). These putative glycan crosslinks would not be detected using typical EXT network extraction procedures (i.e., anhydrous HF deglycosylation), but may nonetheless be involved in and important for EXT crosslinking (just not responsible for wall insolubilization *per se*). These crosslinks may be more important for wall association of NCL EXTs. One approach to address this is to continue to analyze wall preparations using advanced LC–MS/MS techniques that are able to predict and detect crosslinked EXT peptides, such as that described by Gainey (2020). Coupling these studies with *in vitro* studies that utilize synthetic glycoproteins as in Held et al. (2004) may help identify additional and novel crosslinks within the EXT network.

## Author Contributions

MH, AF, and LT helped design, conceive, and outline this manuscript. MH, AF, LT, JM-E, YZ, AS, and MO contributed to the writing of this manuscript. All authors contributed to the article and approved the submitted version.

### Conflict of Interest

The authors declare that the research was conducted in the absence of any commercial or financial relationships that could be construed as a potential conflict of interest.
